# Ten-Year Mortality and Cardiovascular Morbidity in the Finnish Diabetes Prevention Study—Secondary Analysis of the Randomized Trial

**DOI:** 10.1371/journal.pone.0005656

**Published:** 2009-05-21

**Authors:** Matti Uusitupa, Markku Peltonen, Jaana Lindström, Sirkka Aunola, Pirjo Ilanne-Parikka, Sirkka Keinänen-Kiukaanniemi, Timo T. Valle, Johan G. Eriksson, Jaakko Tuomilehto, for the Finnish Diabetes Prevention Study Group

**Affiliations:** 1 School of Public Health and Clinical Nutrition, Food and Health Research Centre, University of Kuopio, Kuopio, Finland; 2 Diabetes Prevention Unit, Department of Chronic Disease Prevention, National Institute for Health and Welfare, Helsinki, Finland; 3 Department of Public Health, University of Helsinki, Helsinki, Finland; 4 Population Studies Unit, Department of Welfare and Health Promotion, National Institute for Health and Welfare, Turku, Finland; 5 Diabetes Center, Finnish Diabetes Association, Tampere, Finland; 6 Science Center, Pirkanmaa Hospital District, Tampere University Hospital, Tampere, Finland; 7 Institute of Health Sciences, University of Oulu, Oulu, Finland; 8 Health Centre of Oulu, Oulu, Finland; 9 Unit of General Practice, Oulu University Hospital, Oulu, Finland; 10 Oulu Deaconess Institute Research Centre, Oulu, Finland; 11 Department of General Practice and Primary Health Care, University of Helsinki, Helsinki, Finland; 12 Vasa Central Hospital, Vasa, Finland; 13 South Ostrobothnia Central Hospital, Seinäjoki, Finland; Institute of Preventive Medicine, Denmark

## Abstract

**Background:**

The Finnish Diabetes Prevention Study (DPS) was a randomized controlled trial, which showed that it is possible to prevent type 2 diabetes by lifestyle changes. The aim of the present study was to examine whether the lifestyle intervention had an effect on the ten-year mortality and cardiovascular morbidity in the DPS participants originally randomized either into an intervention or control group. Furthermore, we compared these results with a population-based cohort comprising individuals of varying glucose tolerance states.

**Methods and Findings:**

Middle-aged, overweight people with IGT (n = 522) were randomized into intensive intervention (including physical activity, weight reduction and dietary counseling), or control “mini-intervention” group. Median length of the intervention period was 4 years and the mean follow-up was 10.6 years. The population-based reference study cohort included 1881 individuals (1570 with normal glucose tolerance, 183 with IGT, 59 with screen-detected type 2 diabetes, 69 with previously known type 2 diabetes) with the mean follow-up of 13.8 years. Mortality and cardiovascular morbidity data were collected from the national Hospital Discharge Register and Causes of Death Register. Among the DPS participants who consented for register linkage (n = 505), total mortality (2.2 vs. 3.8 per 1000 person years, hazard ratio HR = 0.57, 95% CI 0.21–1.58) and cardiovascular morbidity (22.9 vs. 22.0 per 1000 person years, HR = 1.04, 95% CI 0.72–1.51) did not differ significantly between the intervention and control groups. Compared with the population-based cohort with impaired glucose tolerance, adjusted HRs were 0.21 (95% CI 0.09–0.52) and 0.39 (95% CI 0.20–0.79) for total mortality, and 0.89 (95% CI 0.62–1.27) and 0.87 (0.60–1.27) for cardiovascular morbidity in the intervention and control groups of the DPS, respectively. The risk of death in DPS combined cohort was markedly lower than in FINRISK IGT cohort (adjusted HR 0.30, 95% CI 0.17–0.54), but there was no significant difference in the risk of CVD (adjusted HR 0.88, 95% CI 0.64–1.21).

**Conclusions:**

Lifestyle intervention among persons with IGT did not decrease cardiovascular morbidity during the first 10 years of follow-up. However, the statistical power may not be sufficient to detect small differences between the intervention and control groups. Low total mortality among participants of the DPS compared with individuals with IGT in the general population could be ascribed to a lower cardiovascular risk profile at baseline and regular follow-up.

**Trial Registration:**

ClinicalTrials.gov NCT00518167

## Introduction

Type 2 diabetes mellitus (T2DM) as well as lesser degrees of impaired glucose regulation are known to increase the risk of cardiovascular diseases (CVD), in particular coronary heart disease, peripheral vascular diseases, and stroke [1], [2], [3]. Several lifestyle intervention studies have shown that it is possible to prevent or delay the development of manifest T2DM in individuals with impaired glucose tolerance (IGT) [4], [5], [6], [7], [8], [9], [10]. Furthermore, there is evidence that some drugs used for the treatment of hyperglycemia are able to delay the development of T2DM among high risk groups, i.e. in persons with IGT [11], [12], [13].

However, the ultimate goal of prevention of T2DM is to improve the prognosis and overall quality of life of affected individuals, and with regard to health policy, also to prevent increasing costs of the treatment of T2DM and its complications. There are no randomized lifestyle intervention trials in persons with IGT to show that lifestyle changes would reduce CVD mortality or morbidity. Recently, the 20-year results from the China Da Qing Diabetes Prevention Study [10] showed sustained protection against diabetes but did not give a definite answer to the question whether lifestyle changes are beneficial in terms of CVD morbidity or mortality. Also in the DPS, which was the first individually randomized controlled lifestyle intervention trial, the risk of T2DM was substantially lowered even after discontinuation of active intervention and some improvement was seen in the main CVD risk factors [5], [14], [15]. Furthermore, the greater the lifestyle changes were the lower was the risk of T2DM [5], [9].

The main aim of the present study was to examine whether the lifestyle intervention applied in the DPS had an effect on mortality and CVD morbidity by comparing the mortality and CVD morbidity data of the intensive lifestyle intervention group to control group. In addition, we also present follow-up data for a population-based “native control group” (the FINRISK 1992 cohort) [16], because due to the study design also the control group of the DPS can be considered as a “mini-intervention group”.

## Methods

### The DPS study

The protocol for this trial and supporting CONSORT checklist are available as supporting information; see Checklist S1 and Protocol S1. The DPS is a clinical trial with five participating centers (Helsinki, Kuopio, Turku, Tampere, and Oulu) in Finland. Details on the DPS study design, methods, and procedures have been published previously [5], [14], [17]. Briefly, study participants were recruited mainly by screening of high-risk groups who voluntarily responded to local advertisements. The inclusion criteria were 1) age 40 to 64 years at screening, 2) body mass index (BMI) >25 kg/m^2^ at screening, and 3) the mean value of two 75-g oral glucose tolerance tests (OGTT) in the IGT range based on WHO 1985 criteria [18]. Exclusion criteria included recent (within 6 months) CVD event. The randomization of participants started in 1993 and continued until 1998. At the enrollment visit the study physician wrote the names of eligible participants on the centrally-produced randomization list in consecutive order. The study nurse who was responsible for scheduling the visits did not have access to the list. Randomization was stratified by centre, sex, and the mean 2-hour plasma glucose value (7.8–9.4 mmol/l or 9.5–11.0 mmol/l). The DPS was designed to be large enough to be able to detect a 35% reduction in diabetes incidence with 80% power (beta = 20%) at the 2-tailed 5% significance level (alpha = 5%) [17].

A total of 522 overweight men and women were randomly allocated to one of the two treatment modalities, the intensive diet-exercise counseling group (n = 265, the proportion of women 66%) or the control group (n = 257, the proportion of women 69%). The laboratory personnel were not aware of the group allocation but naturally the participants and staff members involved with the intervention were, so the study was only partly masked. The participants randomized to intensive lifestyle intervention were given individualized counseling by the study nutritionists to achieve the lifestyle goals. They were also advised to increase their level of physical activity, and voluntary physical activity sessions were offered. The lifestyle goals were 1) weight reduction of ≥5%, 2) <30% of the daily energy intake from fat, 3) <10% of the daily energy intake from saturated fat, 4) fiber intake ≥15 grams per 1000 kcal, and 5) moderately intense physical activity ≥30 minutes per day. The control participants were given general health behavior information at randomization [14]. The median length of the active intervention period was 4 years (range 1–6 year).

All participants had an annual OGTT, a medical history, and a physical examination with measurements of height (without shoes), weight (in light indoor clothes), waist circumference (midway between the lowest rib and iliac crest) and systolic and diastolic blood pressure. Serum total cholesterol, HDL-cholesterol and triglycerides were determined from fasting samples using an enzymatic assay method.

### FINRISK 1992 survey

The FINRISK 1992 survey was carried out as a part of the FINMONICA cardiovascular risk factor survey, which is described in detail elsewhere [16]. In brief, a stratified random sample of 8000 people living in three geographical areas in Finland (eastern, south-western and southern Finland), was drawn from the National Population Register. The participants received a postal questionnaire on medical history, socioeconomic background, and health behavior, and an invitation to a clinical examination. Three thousand three hundred people of the original study sample were aged between 45 and 64 years. Of these, 2642 (80%) people who had participated in the original clinical examination were invited to this study. Of them, 2087 (79%) attended the OGTT. Totally, glucose tolerance status and all necessary variables for the analyses were available for 1881 participants.

The OGTT procedure and glucose tolerance classification was based on the WHO 1985 recommendations [18]. Plasma glucose concentration was determined in a central laboratory using a glucose dehydrogenase method (Hoffmann-La Roche, Basel, Switzerland).

Individuals who reported that they have diabetes did not have an OGTT and were classified as known T2DM. Individuals with fasting plasma glucose level ≥7.8 mmol/l or 2 h plasma glucose ≥11.1 mmol/l were classified as having screen-detected type 2 diabetes (ST2DM). Those with 2 h plasma glucose ≥7.8 and <11.1 mmol/l, and fasting plasma glucose <7.8 mmol/l were classified as having IGT.

### Total mortality and cardiovascular morbidity

Data on health status were collected through computerized register linkage to two nationwide health registers: the Hospital Discharge Register and the Causes of Death Register, using the national personal identification number.

Data on all patients discharged dead or alive from all hospitals in Finland have been recorded in a computerized Hospital Discharge Register since year 1968. The diagnoses for hospitalizations have been coded according to the International Classification of Diseases (ICD) version 8 during 1967 to 1986, version 9 during 1987 to 1995, and version 10 since 1996.

Both registers were complete with information until the end of year 2006. End points during follow-up were total mortality, and incident cardiovascular events (fatal and non-fatal), defined as the ICD 8 and 9 codes 401–449, and ICD 10 codes I10–I79. These codes include acute coronary events, coronary heart disease, stroke and hypertensive disease. In both registers the diagnoses are assigned by the physician treating the patient. Diagnostic classification in these registers has been validated against diagnoses derived by myocardial infarction registers, and the concordance was found high [19], [20].

In order to estimate the baseline CVD risk according to the measured risk factors at baseline, we calculated for each study person their 10-year probability to have an CVD-event according to the Framingham risk equation [21].

### Ethical considerations

The study protocols of both the DPS study and FINRISK survey were approved by the ethics committee of the National Public Health Institute in Helsinki, Finland. All study participants provided informed consent prior to the clinical assessments. In the DPS study, 17 participants did not give their consent for the register linkages, and they were thus excluded from this study.

### Statistical methods

Participants were followed up until December 31, 2006, with a median follow-up time of 10.6 years for the DPS trial and 13.8 years for the FINRISK Study cohorts. We calculated mortality and CVD incidence rates separately for the DPS and FINRISK study cohorts, and by glucose tolerance status at the initial examination. In univariate analyses, log-rank test was used to assess differences between the glucose tolerance groups. In multivariate Cox models, the relationship between glucose tolerance at baseline and CVD during the follow-up was further analyzed, taking into account possible confounding factors including age, sex, smoking, history of CVD, systolic blood pressure and total cholesterol. All the analyses were done for the entire study cohorts and after exclusion persons who had had a CVD event before the first examination. Analyses were done with the statistics package Stata version 9.2 [22].

## Results

### Baseline data

The DPS intervention and control groups were similar with regard to the main baseline variables (Table 1). Compared with the FINRISK cohort, proportion of smokers (7% vs. 20%–24%) and proportion of men (32% vs. 41%–55%) was lower. Furthermore, mean levels of systolic blood pressure (p<0.01 for all) and serum total cholesterol (p<0.001, except for the FINRISK diabetic group with p = 0.172) were lower among the DPS study participants compared with the FINRISK groups with disturbances in glucose metabolism.

**Table 1:** Baseline characteristics of the participants in the DPS and FINRISK1992 studies by glucose tolerance.

Study:	DPS	DPS	FINRISK	FINRISK	FINRISK	FINRISK
Glucose tolerance:	IGT (intervention)	IGT (control)	Normal	IGT	ST2DM	T2DM
Number of subjects: n	257	248	1570	183	59	69
Age, years	55.4±7.3	55.0±6.9	53.7±6.0	55.8±5.8	55.9±5.4	55.6±6.3
Sex, % men	34.2	31.5	40.9	50.6	55.2	50.7
BMI, kg/m^2^	31.4±4.6	31.2±4.5	26.8±3.9	29.8±5.2	31.7±6.1	30.5±4.9
Smoking, %	7.0	7.3	20.3	24.0	23.7	14.5
Fasting glucose, mmol/l	6.1±0.8	6.2±0.7	5.4±0.6	6.0±0.6	7.8±2.2	10.4±3.0
2-h glucose, mmol/l	8.9±1.5	8.9±1.5	5.5±1.1	8.8±0.9	14.0±3.8	-
Total cholesterol, mmol/l	5.6±1.0	5.6±0.9	5.9±1.0	6.0±1.1	6.2±1.2	5.8±1.3
HDL cholesterol, mmol/l	1.21±0.31	1.22±0.28	1.42±0.35	1.25±0.31	1.19±0.29	1.14±0.31
Triglycerides, mmol/l	1.69±0.80	1.76±0.76	1.49±0.86	2.17±1.52	2.74±1.82	2.78±1.92
Systolic blood pressure, mmHg	139.6±17.7	136.2±17.4	140.3±19.4	148.8±19.5	150.2±21.1	145.6±20.8
Diastolic blood pressure, mmHg	85.7±9.4	85.6±10.0	84.9±11.0	88.8±11.0	88.3±12.9	85.1±9.8
Drug treatment for lipid abnormalities, %	4.3	6.1	2.3	2.8	1.7	10.1
Drug treatment for elevated blood pressure, %	27.7	31.5	13.5	31.9	37.9	36.8
Baseline CVD*, %	8.2	8.1	9.0	19.7	25.4	34.8
Framingham 10-year CVD-probability, %:	14.3	13.2	14.4	19.4	31.0	29.3

* Values are means±standard deviation unless otherwise noted. Baseline CVD: At least one cardiovascular event (ICD 8 and 9 codes: 401–449; ICD 10 codes : I10–I79) according to the Finnish Hospital discharge register before participation in the DPS or FINRISK studies.

Compared with the normoglycemic people in the FINRISK study, all the other groups (i.e. hyperglycemic people) had elevated triglyceride levels, and lower levels of HDL cholesterol (p<0.001 for all comparisons).

Among the DPS participants, 8.2% in the intervention group and 8.1% in the control group had CVD in the outset of the study. In the FINRISK study cohorts there was an increasing trend in the prevalence of CVD from 9.0% in the normoglycemic group to 34.8% in patients with known T2DM. Thus, the baseline prevalence of CVD in the DPS cohort was at the same level as in the FINRISK normoglycemic group.

The estimated 10-year Framingham CVD risk in both of the DPS cohorts was similar to that in the FINRISK normoglycemic group.

### Total mortality

**Figure 1:**
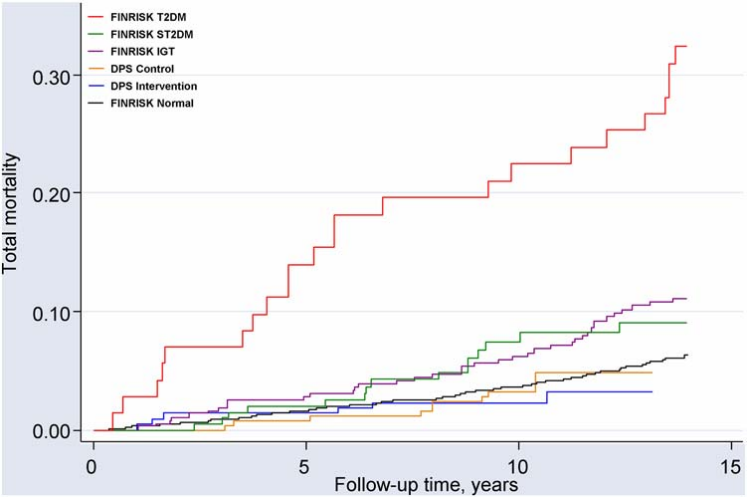
Mortality in the DPS and FINRISK studies. Adjusted for age and sex.

The total number of deaths during the follow-up were 214 (11%) in the FINRISK cohort, and 16 (3%) in the DPS cohort (Table 2). Age- and sex-adjusted Kaplan-Meier estimates of the total mortality are presented in Figure 1. The mortality rates were lowest in the DPS intervention and control groups, being 2.2 and 3.8 per 1000 person-years, respectively (HR = 0.57, 95% CI 0.21–1.58, intervention group of DPS compared to the control group). In the FINRISK cohort, the mortality rates were 6.6, 16.4, 21.0, and 28.8 per 1000 person-years in the normoglycemic, IGT, ST2DM and known T2DM groups, respectively. Compared with the FINRISK IGT group, adjusted HR were 0.21 (95% CI 0.09–0.52) and 0.39 (95% CI 0.20–0.79) for total mortality in the intervention and control groups of the DPS, respectively. The risk of death in the combined DPS cohort (both intervention and control arms) was markedly lower when compared with the FINRISK IGT cohort (adjusted HR 0.30, 95% CI 0.17–0.54)

**Table 2:** Total mortality and cardiovascular events during the follow-up of the DPS and FINRISK studies by glucose tolerance.

Study:	DPS	DPS	FINRISK	FINRISK	FINRISK	FINRISK
Glucose tolerance:	IGT (intervention)	IGT (control)	Normal	IGT	ST2DM	T2DM
Number of persons	257	248	1570	183	59	69
**Total mortality**:						
Person-years, years	2752	2653	20966	2315	713	797
Median follow-up, years	10.6	10.6	13.9	13.8	13.8	13.8
Number of deaths, n	6	10	138	38	15	23
Rate per 1000 person years	2.2	3.8	6.6	16.4	21.0	28.8
95% confidence interval	(1.0–4.9)	(2.0–7.0)	(5.6–7.8)	(11.9–22.6)	(12.7–34.9)	(19.2–43.4)
Hazard ratio (95% CI), unadjusted	0.15 (0.06–0.35)	0.26 (0.13–0.52)	0.40(0.28–0.57)	1.00	1.29(0.71–0.2.37)	1.77(1.05–2.98)
p:	<0.001	<0.001	<0.001	(ref)	0.407	0.032
Hazard ratio (95% CI), adjusted*	0.21 (0.09–0.52)	0.39 (0.20–0.79)	0.52 (0.36–0.74)	**1.00**	1.08 (0.56–2.06)	1.96 (1.15–3.34)
p:	0.001	0.009	<0.001	(ref)	0.824	0.014
**Cardiovascular events**:						
Person-years, years	2488	2452	18878	1855	533	581
Median follow-up, years	10.2	10.2	13.8	13.8	9.5	8.4
CVD events, n	57	54	364	74	33	39
Rate per 1000 person years	22.9	22.0	19.3	39.9	62.0	67.2
95% confidence interval	(17.7–29.7)	(16.9–28.7)	(17.4–21.4)	(31.8–50.1)	(44.0–87.1)	(49.1–91.9)
Hazard ratio (95% CI), unadjusted	0.59 (0.41–0.83)	0.56 (0.40–0.80)	0.48 (0.37–0.62)	1.00	1.58 (1.04–2.39)	1.69 (1.11–2.39)
p:	0.003	0.001	<0.001	(ref)	0.033	0.014
Hazard ratio (95% CI), adjusted*	0.89 (0.62–1.27)	0.87 (0.60–1.27)	0.67 (0.51–0.88)	1.00	1.39 (0.90–2.15)	1.64 (1.02–2.15)
p:	0.528	0.481	0.004	(ref)	0.133	0.042

* adjusted for age, sex, smoke, baseline CVD, systolic blood pressure and total cholesterol.

### CVD morbidity

In the DPS cohort, after a median follow-up time of 10.2 years, there were 57/257 new CVD events in the intervention group and 54/248 in the control group (Table 2). These correspond to the incidence rates of 22.9 and 22.0 per 1000 person-years (HR = 1.04, 95% CI: 0.72–1.51 DPS intervention group compared to the control group). Men and women had a similar CVD incidence whether groups were analyzed separately or combined (data not shown). In addition, no statistically significant differences were found between the intervention and control groups of the DPS with regard to coronary artery angioplasty or by-pass surgery nor drug treatment for dyslipidemia and blood pressure (data not shown).

**Figure 2:**
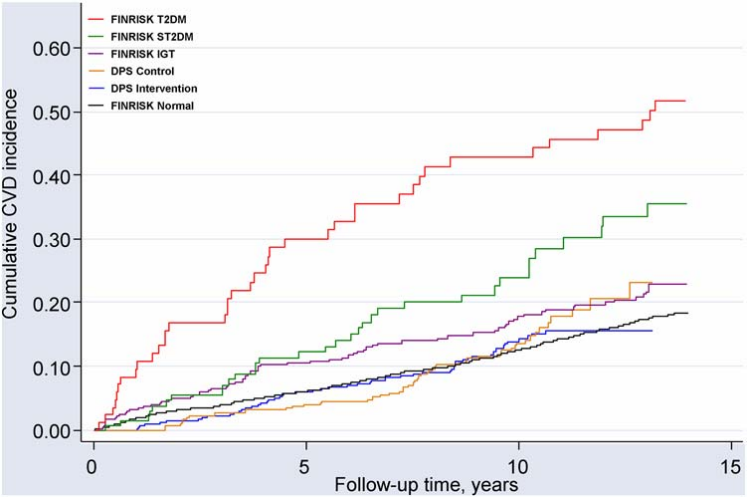
Cumulative incidence of total CVD in the DPS and FINRISK studies. Adjusted for age and sex.

Figure 2 shows the age- and sex-adjusted Kaplan-Meier estimates of cumulative incidence of fatal and non-fatal CVD events. The incidence rates show that the FINRISK known diabetic group had the highest rate of CVD. Both DPS study groups had markedly lower incidence rates compared to the FINRISK IGT group (Table 2). However, there were no statistically significant differences in CVD between the two IGT groups of the DPS study and the FINRISK IGT group after adjustment for baseline risk factors. Hazard ratios for CVD morbidity in the DPS intervention and control groups were 0.89 (95% CI: 0.62–1.27) and 0.87 (95%CI: 0.60–1.27), respectively, compared with the FINRISK IGT group.

We also examined CVD risk after excluding people with a history of CVD at baseline. The analyses showed essentially similar results as presented above for all participants, i.e. the highest CVD risk was found in people with T2DM. Further, in sensitivity analyses, we replicated all the analyses above by excluding early CVD-events, i.e. events occurring during the first 3 years after the baseline health examinations. The results remained essentially unchanged compared with the results presented here (data not shown).

### CVD incidence in relation to main risk factors

In the entire FINRISK cohort, excluding persons with history of CVD, baseline risk factors were strongly associated with CVD incidence (Table 3). When adjusting for age, sex and smoking, all the examined risk factors, with the exception for total cholesterol and lipid medication, remained statistically significant predictors of CVD. Among the DPS participants, the relationship was less clear. Nevertheless, the effects of triglycerides, HDL cholesterol, blood pressure and fasting and 2-h post load plasma glucose levels on CVD risk were quite similar in these two cohorts.

**Table 3:** CVD risk in relation to baseline variables in the DPS and FINRISK studies.

	DPS study				FINRISK study			
	Univariate HR (95% CI)	p	Adj. HR (95% CI)	p	Univariate HR (95% CI)	p	Adj. HR (95% CI)	p
Age	1.34 (1.09–1.63)	0.004			1.57 (1.40–1.75)	<0.001		
Sex (1 men, 0 women)	2.64 (1.72–4.07)	<0.001			1.90 (1.55–2.34)	<0.001		
Smoking (1 yes, 0 no)	2.55 (1.36–4.81)	0.004			1.48 (1.17–1.87)	0.001		
BMI	0.82 (0.64–1.05)	0.110	0.93 (0.72–1.20)	0.576	1.42 (1.30–1.55)	<0.001	1.40 (1.27–1.55)	<0.001
Waist	1.15 (0.89–1.49)	0.288	1.06 (0.80–1.41)	0.683	1.59 (1.44–1.76)	<0.001	1.48 (1.31–1.68)	<0.001
Total cholesterol	0.93 (0.72–1.20)	0.560	0.92 (0.70–1.20)	0.535	1.17 (1.06–1.29)	0.002	1.11 (1.00–1.23)	0.051
Triglycerides	1.46 (1.12–1.89)	0.005	1.44 (1.11–1.86)	0.006	1.31 (1.19–1.43)	<0.001	1.22 (1.09–1.36)	0.001
HDL cholesterol	0.60 (0.44–0.83)	0.002	0.65 (0.47–0.89)	0.007	0.75 (0.67–0.84)	<0.001	0.84 (0.75–1.95)	0.005
Systolic blood pressure	1.27 (1.04–1.55)	0.020	1.19 (0.94–1.52)	0.145	1.46 (1.34–1.59)	<0.001	1.31 (1.19–1.45)	<0.001
Diastolic blood pressure	1.13 (0.90–1.43)	0.287	1.07 (0.84–1.36)	0.598	1.35 (1.23–1.49)	<0.001	1.28 (1.16–1.42)	<0.001
Fasting glucose	1.30 (0.96–1.77)	0.089	1.24 (0.91–1.68)	0.176	1.35 (1.24–1.47)	<0.001	1.28 (1.17–1.40)	<0.001
2-h glucose	1.28 (0.88–1.86)	0.198	1.35 (0.95–1.91)	0.099	1.32 (1.18–1.48)	<0.001	1.25 (1.11–1.41)	<0.001
Lipid medication (1 yes, 0 no)	1.13 (0.37–3.51)	0.829	1.08 (0.38–3.12)	0.884	1.97 (1.94–4.11)	0.070	1.72 (0.82–3.62)	0.153
Blood pressure medication (1 yes, 0 no)	1.82 (1.16–2.84)	0.009	1.80 (1.12–2.89)	0.015	2.33 (1.83–2.97)	<0.001	2.11 (1.65–2.71)	<0.001

Univariate HR: Each of the variables listed, respectively, is included in a model alone.

Adj. HR: Each of the variables listed, respectively, is included in a model together with age, sex and smoking.

Excluding individuals who had CVD before participation to the study. For continuous variables, the hazard ratio is given per 1 standard deviation unit.

When all the variables in Table 3 were simultaneously considered in a stepwise multivariate selection procedure, age, sex, smoking and HDL cholesterol remained statistically significant predictors of CVD in the DPS cohort. Additionally including the DPS treatment group into the model did not improve prediction. In the FINRISK cohort, besides age, sex and smoking, also systolic blood pressure, waist circumference and medication for high blood pressure were statistically significant predictors of CVD.

Finally, we analyzed the effects of changes in selected risk factors within the first year of the DPS study on the CVD risk (Table 4). In unadjusted analyses, changes in body weight and diastolic blood pressure were statistically significant predictors, but after further adjustment for age, sex and smoking, only change in diastolic blood pressure remained as statistically significant predictor of CVD in a way that reduction of diastolic blood pressure diminished the risk of CVD.

**Table 4:** Changes in CVD risk factors and their relation to CVD events during follow-up in the DPS.

	Change from baseline to year 1		Hazard ratio per 1 standard deviation change			
	DPS intervention	DPS control	Intervention and control groups combined			
	mean±standard deviation	mean±standard deviation	Univariate HR (95% CI)	p	Adj. HR (95% CI)	p
Weight	−4.5±5.0	−1.0±3.7	1.27 (1.02–1.59)	0.032	1.22 (0.94–1.58)	0.144
Waist	−4.3±5.2	−1.4±4.9	1.12 (0.90–1.41)	0.317	1.10 (0.87–1.40)	0.417
Total cholesterol	−0.12±0.73	−0.10±0.72	0.84 (0.66–1.08)	0.181	0.87 (0.68–1.11)	0.258
Triglycerides	−0.19±0.56	−0.02±0.67	1.00 (0.80–1.26)	0.975	1.06 (0.85–1.33)	0.583
HDL cholesterol	0.05±0.19	0.02±0.17	1.05 (0.84–1.30)	0.672	1.05 (1.05–1.32)	0.696
Systolic BP	−5.2±14.3	−1.5±14.7	1.16 (0.93–1.44)	0.203	1.22 (0.98–1.52)	0.072
Diastolic BP	−4.7±8.6	−2.8±9.5	1.25 (1.02–1.53)	0.028	1.31 (1.07–1.59)	0.007
Glucose, 0 h	−0.23±0.68	0.04±0.66	1.23 (0.93–1.64)	0.146	1.22 (0.90–1.66)	0.192
Glucose, 2 h	−0.80±1.86	−0.28±2.18	1.24 (0.95–1.63)	0.120	1.20 (0.92–1.58)	0.177

Univariate HR: Each of the variables listed, respectively, is included in a model alone.

Adj. HR: Each of the variables listed, respectively, is in included in a model together with age, sex and smoking.

Excluding individuals who had CVD before participation into the study.

## Discussion

Our main aim was to examine whether a lifestyle intervention with weight reduction, dietary counseling and increasing physical activity could reduce total mortality and CVD morbidity in the Finnish DPS. The CVD morbidity was identical for the DPS intervention and control groups. Thus, we found no evidence of overall CVD risk modification by the DPS lifestyle intervention in persons with IGT. In keeping with findings from numerous previous prospective studies [1], [2], [3], both IGT and T2DM in the FINRISK cohort increased the risk of CVD.

Interestingly, total mortality in both groups of the DPS participants was markedly lower when compared with the FINRISK participants with IGT. The mortality data, however, should be interpreted with caution due to a small number of cases and as the difference could reflect the distinct selection procedures of the study populations, leading to divergent risk profiles as shown by differences in the risk factor levels and the calculated Framingham 10-year CVD-probability. However, besides these factors, also the regular follow-up and health consciousness of the DPS study participants could explain the low mortality among them.

The incidence rates of T2DM in the DPS study during the overall follow-up of 7 years were 4.3 and 7.4 cases per 100 person-years in the intervention and control groups, respectively (hazard ratio 0.57, 95% CI 0.44 to 0.79, p<0.001) [9]. Whereas there is firm evidence that lifestyle changes prevent T2DM in well controlled long-term trials [4], [5], [6], [7], [8], [9], [10], little is known whether a lifestyle intervention could modify CVD mortality or morbidity in such trial settings. Recently, 20-years follow-up results from the Chinese Da Qing Diabetes Prevention Study showed no statistically significant differences in CVD morbidity or mortality between the intervention and control groups, although a non-significant 17% reduction was seen in CVD mortality in the combined (diet and/or physical activity) intervention group [10]. Yet, the incidence of T2DM was 43% lower in the intervention group compared with the control group after 20-years of follow-up. In the non-randomized Malmö Preventive Trial with a 12-year follow-up of men with IGT, both the over-all and ischemic heart disease mortality were lower among those who were participating in the diet and exercise intervention group, compared with routine treatment group [23], but due to the non-randomized study design these results should be considered with precaution.

The DPS was not initially planned to examine the effect of lifestyle intervention on total mortality or CVD morbidity. Therefore, the statistical power may not be sufficient to detect small differences in CVD events between the intervention and control groups in the DPS. Another explanation for the equal CVD risk in the lifestyle intervention and control groups in the DPS may be that lifestyle changes were not extensive enough to result in significant CVD risk reduction even though they were sufficient to reduce the rate of progression to T2DM. Nevertheless, in addition to the improvement in glycemia, some beneficial changes were achieved in other risk factors of CVD in the DPS [5], [9], [15]. Importantly, both the intervention and control group participants in the DPS were regularly examined by the study physician. There was no difference in drug treatment for elevated serum lipids or blood pressure, and also the number of invasive treatments for coronary artery disease was similar in both groups. The DPS participants irrespective of the randomization group were well motivated and carefully monitored in terms of their health status, and they also had more favorable risk profile compared with the FINRISK ‘true control cohort’. Thus, we suggest that they represented the healthier proportion of persons with IGT, and this may explain why no significant effects on CVD morbidity could be observed by lifestyle intervention. This view is strongly supported by their low mortality rates irrespective of the randomization group when compared to FINRISK control persons. However, the present results do not exclude the possibility that a study design targeted a priori at CVD prevention in people at high risk for T2DM by lifestyle modification as applied in the DPS could have an effect on CVD morbidity. Furthermore, a longer follow-up period may be needed to obtain a definite answer to this question.

As in many previous studies, low HDL-cholesterol and high triglycerides showed to be the main risk factors for CVD in both persons with IGT and T2DM [1], [2], [24]. Weight reduction and increase in physical activity are the most important lifestyle factors to improve these lipid abnormalities. Recent results of the treatment of very obese people by bariatric surgery show that weight reduction indeed may improve the prognosis of obese people, and not only T2DM risk but also total mortality was reduced [25]. Suggestive evidence to this direction was also observed in the present study; BMI was a risk factor for CVD only in the FINRISK cohort, with no active intervention in this regard. Furthermore, in univariate analyses on the DPS study persons 1-year weight loss seemed to reduce cardiovascular risk among IGT persons free of CVD at baseline, with 5 kg weight reduction resulting in 27% CVD risk reduction; however, the effect was no longer significant in analysis adjusted for age, sex, and smoking. Also the early results from the US Diabetes Prevention Program showed improvement in CVD risk factors after intensive lifestyle intervention compared with the placebo or metformin treatment, but no difference in CVD events between the groups was observed after 3 years follow-up [26].

In conclusion, the DPS lifestyle intervention that resulted in marked decrease in the incidence of T2DM even after the active intervention period did not decrease the risk of CVD during a 10-year follow-up. A lower initial CVD risk profile and regular follow-up may explain the observed lower mortality among the DPS participants when compared to the population based IGT or normoglycemic cohorts. In the future, lifestyle intervention studies to prevent T2DM should be focused on individuals, who besides an increased risk of T2DM also have a markedly increased CVD risk. The future studies should also be sufficiently powered to detect modest differences in CVD morbidity or mortality. Finally, meta-analyses of multiple intervention trials may offer an opportunity to solve the question on the effects of lifestyle changes on CVD morbidity and mortality among persons with IGT.

## Supporting Information

Protocol S1 Trial Protocol
(0.05 MB DOC)

Checklist S1 CONSORT checklist
(0.06 MB DOC)
