# Supplementary material for: Ten-Year Mortality and Cardiovascular Morbidity in the Finnish Diabetes Prevention Study—Secondary Analysis of the Randomized Trial
Source: PLoS One. 2009 May 21;4(5):e5656. doi: 10.1371/journal.pone.0005656 (PMC2682577; doi:10.1371/journal.pone.0005656)
Supplement: Protocol S1 — Trial Protocol (0.05 MB DOC) [file pone.0005656.s001.doc]

#

**The Finnish Diabetes Prevention Follow-up Study :**

**A follow-up study on the effect of a dietary and exercise intervention in the prevention of diabetes and its vascular complications.**

**MANUAL OF OPERATIONS**

**4.12.2002**

**1. Objectives and methods**

The Diabetes Prevention Follow-up Study has the following major purposes:

**Aim 1.** To evaluate the effect of intervention on the risk of type 2 diabetes and components of the metabolic syndrome by determining:

**1.1** The **factors (changes) that are independently related to the prevention of the progression from IGT to diabetes** (body size, diet, physical activity) and to determine the importance of their interaction on the outcome

**1.2** **The factors that are related to the worsening of glucose tolerance in the control group** in order to obtain new data on **natural history of type 2 diabetes.**

**Aim 2.** To carry out a **prospective follow-up in order to determine the long-term effects** of the intervention programme on lifestyle patterns, on the worsening of glycaemia, on the long-term incidence of diabetes and on the metabolic syndrome.

**Aim 3.** To **determine the effect of intervention on the cardiovascular risk score and to determine the incidence of late complications related to hyperglycaemia** by standardised clinical assessments and by monitoring fatal and non-fatal coronary and stroke events in the two randomisation groups.

The intervention study (DPS) will be completed, according to the recommendation of the end-point committee, at the next annual visit of each study subject, by spring-2001. The length of the intervention will therefore vary between individuals. The DPS Follow-up Study will start one year after each subject’s last DPS-visit.

**2. Study subjects**

The study population consists of the subjects who were randomized into the DPS-study, excluding the ones who have expressed their unwillingness to continue the study. The subjects will be asked to sign an informed consent form at their first annual visit.

The subjects with diagnosed diabetes mellitus will have all other measurements taken except for the oral glucose tolerance test (fasting plasma glucose and serum insulin will be measured).

**4. Methods**

The subjects will get a mailed invitation to the study clinic. They will be asked to fill in the 3-day food diary (sunday-monday-tuesday or thursday-friday-saturday) and 24-h exercise diary (working day: monday-friday) before the visit (other forms may also be sent to the subjects or they may be filled during the OGTT). **All the forms are checked for completenes** at the visit.

The subjects are asked to fast for 12 hours before the visit, and they are emphasized to eat normally (typical amounts of fat and carbohydrate containing foods) for 3 days before the visit (dieting etc. will affect the OGTT results). Heavy exercise on the day before the visit is forbidden (in that case the visit is rescheduled). Acute illness is also a reason to reschedule the visit.

The visit time window is the date of the original annual DPS visit ± 3 months.

**Follow-up**

The following data will be collected at annual visits during the follow-up study:

| **Biochemical parameters** | **Anthropometric parameters** | **Physiological parameters** | **Quality of life** | **Life-style** | **Complications** |
| --- | --- | --- | --- | --- | --- |
| OGTT * plasma glucose: fasting, 30min, 1h, and 2h | weight | blood pressure: systolic and diastolic | RAND-36 Item Health Survey | KIHD 24-Hour Total Physical Activity Record | fundus photography |
| OGTT * serum insulin: fasting, 30min, and 2h | waist circumference | heart rate | BDI -Beck Depression Inventory | KIHD 12-month Leisure Time Physical Activity History | neuropathy assessment |
| serum total cholesterol | hip circumference | 2-km walking test |  | 3-day food record | WHO Cardiovascular Questionnaire |
| serum HDL cholesterol | sagittal abdominal diameter |  |  | study questionnaire | peripheral pulses |
| serum triglycerides | horizontal abdominal diameter |  |  |  | urinary albumin |
| serum creatinine | fat mass |  |  |  | urinary creatinine |
| serum uric acid | lean body mass |  |  |  | ECG |
| HbA1c |  |  |  |  | hospital discharge register |
| serum fibrinogen** |  |  |  |  | death register |
| serum PAI-1** |  |  |  |  |  |
| serum c-peptide*** |  |  |  |  |  |
| GAD-antibodies |  |  |  |  |  |
| genetic markers |  |  |  |  |  |

** fibrinogen and PAI-1 every three years

*** c-peptide in those who get DM

**Laboratory determinations**

Plasma glucose will be determined locally according to the standard guidelines. All biochemical parameters will be analysed at the laboratory of National Public Health Institute in Helsinki to avoid any biases between centres. After processing the samples locally they will be stored at -20°C and transported to the central biochemical laboratory.

In subjects who developed diabetes during the study we will determine serum C-peptide levels at annual follow-up visits to determine the β-cell function. In all subjects we have determined GAD-antibodies. Thus, we will be able to find out if some of the diabetic patients have type 1 diabetes process.

**Genetic factors**

Several currently known genetic markers suggested for obesity or diabetes have already been determined in all subjects, such as PPR1_γ_, _β_3AR, UCP_1_, UCP_2_, NPY, leptin. All subjects developing diabetes and a random sample of 150 non-diabetic subjects will be HLA genotyped for class I and class II loci. Additional genotyping to be done during the study will include new markers for type 2 diabetes that will be found during the ongoing genome scans, genes related to dyslipidemia and hypertension. Individual and joint effects of various candidate genes, and their interaction with environmental risk factors for type 2 diabetes and metabolic syndrome will be evaluated.

# **Assessment of physical activity**

Leisure time physical activity will be assessed annually using the Kuopio Ischaemic Heart Disease Risk Factor Study (KIHD) 12-Month Leisure Time Physical Activity History. Total physical activity was and will be assessed using the KIHD 24-Hour Total Physical Activity Record.

## **Collection and diagnostic classification of complications of IGT and type 2 diabetes**

Data on the complications of IGT and type 2 diabetes, including acute coronary events, cerebrovascular disease, peripheral vascular disease, nephropathy, neuropathy, retinopathy, cataract, and deaths, are collected prospectively from hospitals by computer linkage to the national hospital discharge register and death register using the unique personal identification code given to all Finnish citizens and using the ICD- codes.

**Coronary events.** The diagnostic classification of coronary events is identical to that of the FINMONICA acute myocardial infarction (AMI) register [23]. Hospital documents, death certificates, autopsy reports and medico-legal reports are used as sources of information. The diagnostic classification of coronary events is based on symptoms, ECG, cardiac enzyme elevations, autopsy findings and history of coronary heart disease (CHD). Each suspected coronary event (ICD-10 codes I20-I25) is classified into 1) definite acute myocardial infarction (AMI), 2) probable AMI, 3) prolonged chest pain episode, 4) ischemic cardiac arrest with successful resuscitation. Definite AMIs, probable AMIs and prolonged chest pain episodes are used as outcome events.

**Cerebrovascular events.** The diagnostic classification of strokes is identical to that of the FINMONICA stroke register [24]. Hospital documents, death certificates, autopsy reports and medico-legal reports are used as sources of information. The diagnostic classification of strokes is based on symptoms, clinical findings, computed tomography (CT) or magnetic resonance imaging

(MRI) findings and autopsy findings. Each suspected ischaemic (ICD-10 codes I33-I34) or haemorrhagic (ICD-10 codes I30-I31) stroke is classified into 1) definite stroke, 2) transient ischaemic attack (TIA) or 3) unclassifiable. Definite strokes are used as primary outcome events.

**Other complications.** The clinical diagnosis of peripheral vascular disease, nephropathy, neuropathy, retinopathy, cataract and erectile dysfunction and the cause of death are ascertained from hospital documents, death certificates, autopsy reports and medico-legal reports. Deaths are classified into cardiovascular deaths (ICD-10 codes I00-I99). Death codes were all validated according to the international criteria.

**Fundus photography**

One macula-centered 45º red-free black-and-white fundus photograph of both eyes of each participant are taken with Canon CN 45 (non mydriatic) camera using Tmax 100 black and white film and red-free light using a green absorption filter (Kodak Wratten 58). Pupils are dilated for photography with tropicamide drops. Paper enlargements measuring 10 x 12 cm are obtained.

**Microalbuminuria**

Milligrams of albumin that are excreted per mmols of creatinine are measured from a first morning urine specimen (4-6 hours in the bladder) which is collected at the morning of the laboratory visit. Urine specimen (1 dl) is collected at home and kept in the fridge until it is taken to the laboratory. In the laboratory urine is mixed, poured into a plastic tube and frozen (-20 C).

1. **Performers of the research and resources**

The study will be performed in 5 study centres: in Helsinki, Kuopio, Turku, Tampere and Oulu.

The study centres will take responsibility to conduct the follow-up of the study subjects: the annual laboratory and other measurements and collection of the exercise, dietary, and other questionnaire data. In addition, certain centres will have special responsibilities. Kuopio centre has expertise on genetic issues and will coordinate the genetic sample collection and analyses together with Helsinki. Turku centre will take responsibility in the laboratory analyses of the haemostatic factors. Helsinki centre will take care of the over-all coordination of the multicentre study, biochemistry and ECG coding, and handles the data management and statistical analyses of the data. Each centre's staff will include a study nurse, nutritionist and physician and additional personnel as appropriate.

**Study group:**

**Helsinki:**

Johan Eriksson, MD, PhD, docent, head of the local DPS centre

Timo Valle, MD, physician

Jaakko Tuomilehto, MD, PhD, professor

Jaana Lindström, MSc, nutritionist

Zygimantas Cepaitis, MSc, data manager

Paula Summanen, MD, PhD, ophthalmologist

Gunvor C. von Wendt, MD, PhD, ophthalmologist

**Kuopio:**

Matti Uusitupa, MD, PhD, professor, head of the local DPS centre

Anne Louheranta, MSc, nutritionist

Martti Hakumäki, MD, PhD, physician

Timo Lakka, MD, PhD

**Turku:**

Helena Hämäläinen, MD, PhD, head of the local DPS centre

Sirkka Aunola, PhD

Merja Rastas, MSc, nutritionist

**Tampere:**

Pirjo Ilanne-Parikka, MD, head of the local DPS centre

Virpi Salminen, MSc, dietician

Pirjo Lehto, MSc, physiotherapist

**Oulu:**

Sirkka Keinänen-Kiukaanniemi, MD, PhD, professor, head of the local DPS centre

Mauri Laakso, MD, physician

Marjo Mannelin, MSc, dietician

**3. Study visits**

The study subjects will have one annual visit to the study clinic. They will be mailed an invitation, with the food and exercise diary forms and instructions how to fill them (a portion size picture booklet is also mailed). The first follow-up visit (one year after the termination of the DPS) is visit number 1F and the second 2F, etc. If the subject is a drop-out or has been diagnosed with DM, the 1F-visit will be scheduled one year after the date the last DPS-visit would have been done assuming that the subject had been in the study until the end of the follow-up (example: 0-year DPS visit 1.6.1997; DM diagnosed 1.6.1999 at 2-year visit; end of the DPS would otherwise have been in June 2000; the 1F-visit will be in June 2001).

**Diabetic complications are assessed with annual fundus photography, neuropathy assessment, peripheral pulses, urinary albumin and urinary creatinine.**

**Annual laboratory visits**

- **informed consent form (at first follow-up visit)**
- **fasting venous blood specimens**
- **3 serum samples**
- **2 tubes for analyses of s-chol, s-HDL-chol, s-trigl, s-creatinine, s-uric acid (serum c-peptide for those with DM diagnosis)**
- **5 tubes for storage**
- **fasting insulin sample (serum)**
- **fasting glucose sample (plasma)**
- **EDTA blood sample for HbA1c**
- **OGTT: 75g glucose**
- **30min, 1h and 2h plasma samples for glucose**
- **30min and 2h serum samples for insulin**
- **WHO cardiovascular questionnaire (nurse/nutritionist completes)**
- **Annual Questionnaire**
- **15D**
- **dietary assessment: 3-day food diary (nutritionist)**
- **physical activity assessment: 24hr and 12months**
- **blood pressure**
- **pulse**
- **weight**
- **lean body mass (using Futrex^R^ or other method, if available)**
- **hip and waist circumference**
- **sagittal and transverse diameters**
- **ECG**

**If glucose values are diabetic: fasting p-gluc ≥7.8 or 2-hour p-gluc > 11.0, the subject is asked for the confirmatory OGTT (minimum of 1wk interval). In the confirmatory OGTT only fasting and 2-h plasma glucose values will be obtained and measured. The confirmatory OGTT will not be done it the subject has already been diagnosed with diabetes during the DPS-study or the follow-up.**

Laboratory results are mailed to the subject; if some value is outside the reference area the subject is asked to contact his/hers own physician.

**The 2km walking test is performed by all subjects annually or every two years.**
